# Comparing Multimorbidity Patterns Among Discharged Middle-Aged and Older Inpatients Between Hong Kong and Zurich: A Hierarchical Agglomerative Clustering Analysis of Routine Hospital Records

**DOI:** 10.3389/fmed.2021.651925

**Published:** 2021-07-21

**Authors:** Francisco T. T. Lai, Patrick E. Beeler, Benjamin H. K. Yip, Marcus Cheetham, Patsy Y. K. Chau, Roger Y. Chung, Eliza L. Y. Wong, Eng-Kiong Yeoh, Edouard Battegay, Samuel Y. S. Wong

**Affiliations:** ^1^The Jockey Club School of Public Health and Primary Care, Faculty of Medicine, The Chinese University of Hong Kong, Hong Kong, China; ^2^Centre for Safe Medication Practice and Research, Department of Pharmacology and Pharmacy, Li Ka Shing Faculty of Medicine, The University of Hong Kong, Hong Kong, China; ^3^Department of Internal Medicine, University Hospital Zurich and University of Zurich, Zurich, Switzerland

**Keywords:** comorbidity, machine learning, multiple chronic conditions, non-communicable disease, population aging

## Abstract

**Background:** Multimorbidity, defined as the co-occurrence of ≥2 chronic conditions, is clinically diverse. Such complexity hinders the development of integrated/collaborative care for multimorbid patients. In addition, the universality of multimorbidity patterns is unclear given scarce research comparing multimorbidity profiles across populations. This study aims to derive and compare multimorbidity profiles in Hong Kong (HK, PRC) and Zurich (ZH, Switzerland).

**Methods:** Stratified by sites, hierarchical agglomerative clustering analysis (dissimilarity measured by Jaccard index) was conducted with the objective of grouping inpatients into clinically meaningful clusters based on age, sex, and 30 chronic conditions among 20,000 randomly selected discharged multimorbid inpatients (10,000 from each site) aged ≥ 45 years. The elbow point method based on average within-cluster dissimilarity, complemented with a qualitative clinical examination of disease prevalence, was used to determine the number of clusters.

**Results:** Nine clusters were derived for each site. Both similarities and dissimilarities of multimorbidity patterns were observed. There was one stroke-oriented cluster (3.9% in HK; 6.5% in ZH) and one chronic kidney disease-oriented cluster (13.1% in HK; 11.5% ZH) in each site. Examples of site-specific multimorbidity patterns, on the other hand, included a myocardial infarction-oriented cluster in ZH (2.3%) and several clusters in HK with high prevalence of heart failure (>65%) and chronic pain (>20%).

**Conclusion:** This is the first study using hierarchical agglomerative clustering analysis to profile multimorbid inpatients from two different populations to identify universalities and differences of multimorbidity patterns. Our findings may inform the coordination of integrated/collaborative healthcare services.

## Introduction

Multimorbidity is commonly referred to as the co-occurrence of two or more chronic health conditions (1) and is consistently associated with poorer quality of life (2), more healthcare utilization (3), deteriorating mental health (4), and greater risk of mortality (5). Various models of care have been proposed and trialed to address this complexity in clinical practices (6, 7).

Multimorbid patients are clinically diverse (8) and may have markedly different prognoses due to different disease combinations (9). Such heterogeneity limits the provision of integrated or collaborative care in various healthcare settings (10, 11) as evidence-based clinical guidelines are inadequate (12) given the scarcity of randomized controlled trials conducted on multimorbid populations (13), even those with more prevalent disease combinations (14). This challenge is often further complicated by the need to manage multiple drug regimens (polypharmacy) (15) and the associated adverse effects (16).

Numerous attempts have thus been made to identify common patterns of multimorbidity to simplify the problem (8, 17). Prados-Torres et al. (17) identified 97 different combinations of two or more co-occurring chronic conditions, which were mostly represented as cardiometabolic, musculoskeletal, and mental patterns. In a more recent review, Ng et al. (8) updated the literature search and evaluated the methods by which multimorbidity patterns are identified. Clustering analysis has been found to be the commonest approach to identifying disease patterns: grouping together similar diseases that are found in the same individuals (8, 17). While this approach omits the possibility that one disease may belong with more than one cluster and that even individuals with the same disease may be clinically distinct from each other given other conditions, very few reviewed studies (18) adopted clustering analysis or other methods to group similar patients instead of similar diseases (8, 17). Furthermore, very few studies have compared multimorbidity patterns across countries using the same methods (19). Similarities and dissimilarities between multimorbidity patterns observed in different contexts, therefore, remain unexplored. In fact, the presence of universal patterns may strengthen the rationale for more randomized controlled trials to be conducted on multimorbid patients because results would confer cross-country implications.

This study aims to describe and compare the patterns of co-occurring chronic conditions among discharged multimorbid inpatients in Hong Kong and Zurich using a hierarchical agglomerative clustering analytic approach, which is typically used for grouping similar individuals based on predetermined ranges of characteristics. Since the healthcare system as well as the cultural and demographic characteristics differ drastically between the two sites, observations of similar multimorbidity patterns may potentially imply universal challenges facing clinicians globally.

## Materials and Methods

### Study Design and Data Collection

We conducted a retrospective analysis of clinical records of discharged patients aged ≥ 45 from all public hospitals in Hong Kong (representing >90% of all inpatient services) during January 2010-December 2013 and from the University Hospital Zurich (general acute hospital, teaching hospital for University of Zurich) during August 2009-August 2017. The discrepancy of observation period was mainly due to the much smaller amount of data generated from Zurich as it is only one hospital compared with the whole public hospital system in Hong Kong.

The data contained information on patients' age, sex, the length of stay, and the first 15 clinical diagnoses made during the hospital stay coded with the International Classification of Diseases, Ninth Revision (ICD-9) for Hong Kong and with the Tenth Revision (ICD-10) for Zurich. Multimorbidity is defined as having two or more chronic conditions using a list of 30 diseases coded either by ICD-9 or ICD-10. The list of chronic conditions is based on validated coding algorithms summarized by Tonelli et al. (20) and included alcohol misuse, asthma, atrial fibrillation, chronic heart failure, chronic kidney disease, chronic pain, chronic pulmonary disease, chronic viral hepatitis B, cirrhosis, dementia, depression, diabetes, epilepsy, hypertension, hypothyroidism, inflammatory bowel disease, irritable bowel syndrome, lymphoma, metastatic cancer, multiple sclerosis, myocardial infarction, non-metastatic cancer (breast, cervical, colorectal, lung, and prostate), Parkinson's disease, peptic ulcer disease, peripheral vascular disease, psoriasis, rheumatoid arthritis, schizophrenia, severe constipation, and stroke or transient ischemic attack (TIA). These diseases are all convertible between ICD-9 and ICD-10 in accordance with the Tonelli algorithms and therefore diseases could be mapped between the sites. Ten thousand patients with two or more of these conditions from each site were randomly selected and included with the “sample” function according to approach described by Ripley (21) in R, version 3.6.0 (R Foundation for Statistical Computing, Vienna, Austria). This sample size was approximately four times the minimum required number recommended for a clustering analysis of 32 variables (22).

30-day readmission and length of stay in hospital were measured to compare health care utilizations between clusters and sites. We considered only the first discharge of each patient during the data collection period to observe their clinical profiles and length of stay, then followed them up 30 days after their baseline discharge to observe any readmission.

### Clustering Analysis

Stratified by sites (Hong Kong and Zurich), a hierarchical agglomerative clustering analysis implemented with R package “hclust” was conducted to form clusters of patients starting by grouping similar individuals in terms of age groups (categorized according to the World Health Organization's 5-year intervals: 45-49, 50-54, 55-59, 60-64, 65-69, 70-74, 75-79, 80+), sex, and the presence of listed chronic conditions with dissimilarity between patients measured by Jaccard index (23). Using the Ward's method, the pair of clusters or patients merged in each step was the one associated with the smallest increase in the total within-cluster dissimilarity. To inform our decision on the number of clusters to be specified, we first plotted the total within-cluster dissimilarity by number of specified clusters to look for an elbow point at which total within-cluster dissimilarity cease to decrease significantly, then we conducted a qualitative examination of the disease prevalence across clusters to eventually determine the optimal number of clusters from a clinical perspective, i.e., the balance between interpretability and meaningful clinical grouping of patients. Clustering of patients was then compared between sites.

We performed statistical analyses with R and there were no missing data in the anonymized hospital records. An ethics waiver has been granted by Cantonal Ethics Committee of Zurich for the analysis of Zurich inpatient data (Ref: NZ-B-Nr.2017-00882) while the analysis of Hong Kong inpatient data was approved by the Survey and Behavioral Ethics Committee of the Chinese University of Hong Kong (Project Code: Elderly Care – CUHK). As only secondary analysis of anonymized inpatient data was performed, no informed consent was required.

## Results

**Figure 1:**
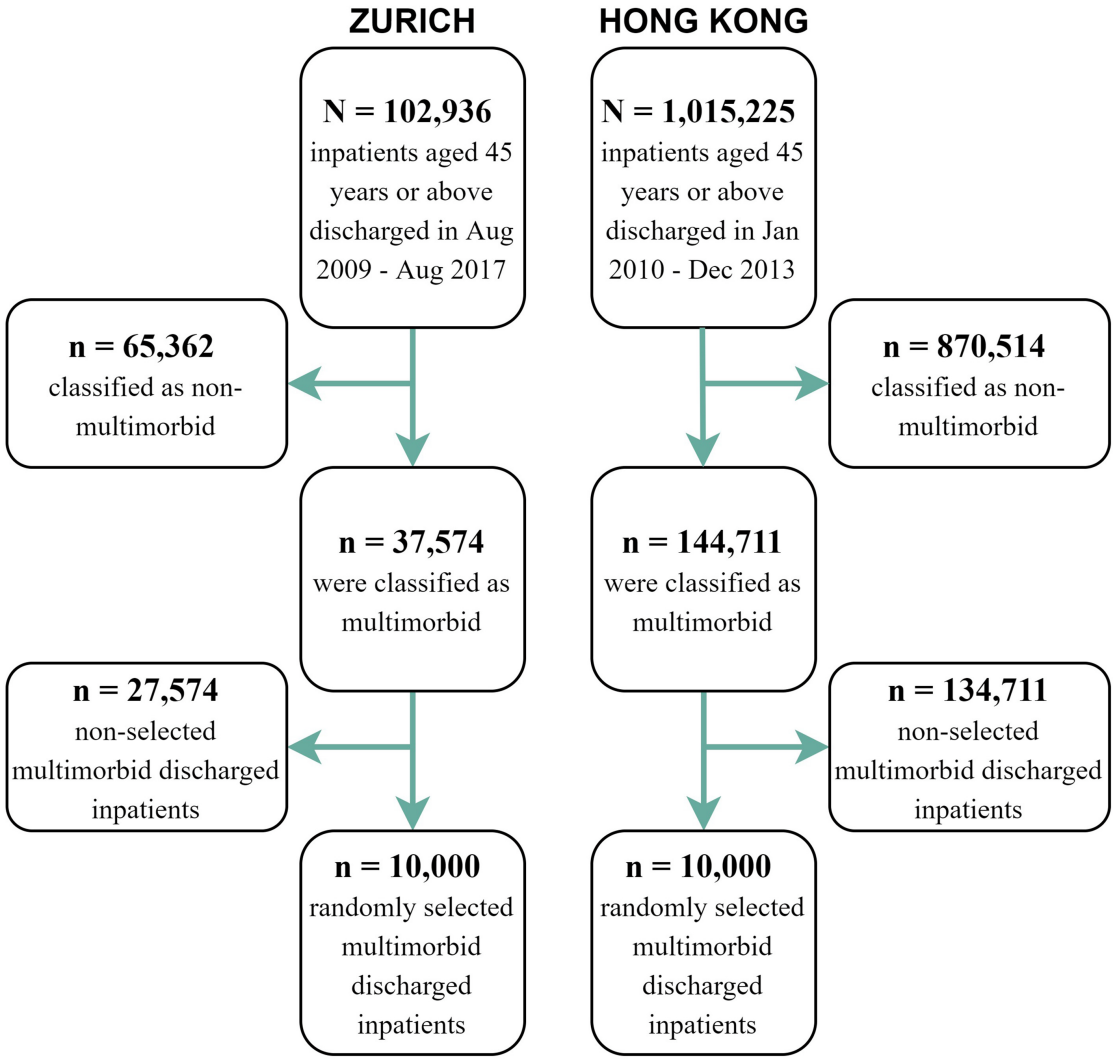
Graphical representation of the sample selection for the analysis.

Over the corresponding study periods (see Figure 1), there were 1,015,225 inpatients aged ≥ 45 discharged from Hong Kong public hospitals among which 144,711 were multimorbid; and of the 102,936 discharged from the University Hospital Zurich, 37,574 were multimorbid. For each site, 10,000 patients were randomly selected for analysis.

### Comparison of Disease Prevalence and Demographics

Table 1 shows the comparison of sample characteristics and disease prevalence between sites. First, the sample from Hong Kong had an older median age (75 vs. 70) and fewer males (51.2 vs. 57.6%) than the Zurich sample. Second, while 30-day readmission rate was similar, the median length of current stay is substantially greater in Zurich (7 vs. 4 days). Third, patients from Zurich had more diagnoses than those from Hong Kong, where only 1.4% of the sample had five or more diseases, compared with 5.0% in Zurich. There were notable differences in specific disease prevalence between the sites. Prevalence of alcohol misuse, epilepsy, and cancer prevalence among patients in Zurich was about triple that in Hong Kong. Much fewer Zurich patients had atrial fibrillation and heart failure compared with Hong Kong patients and there was a sharp contrast in peripheral vascular disease prevalence (0.0% in Hong Kong vs. 10.4% in Zurich).

**Table 1:** Comparison of demographics and disease prevalence between sites.

	**Hong Kong**	**Zurich**	
***N***	**10,000**	**10,000**	***P*-value**^**a**^
Age (median [IQR])	75 [64, 82]	70 [61, 78]	<0.001
Male (%)	5,125 (51.2)	5756 (57.6)	<0.001
Length of current stay (median [IQR])	4 [2, 9]	7 [3, 13]	<0.001
30-day readmission (%)	1,034 (10.3)	1,185 (11.8)	0.001
Number of chronic diseases (%)			<0.001
Two	6,727 (67.3)	5,461 (54.6)	
Three	2,503 (25.0)	2,865 (28.6)	
Four	635 (6.3)	1,171 (11.7)	
Five or more	135 (1.4)	503 (5.0)	
**Chronic diseases (%)**			
Alcohol misuse	176 (1.8)	650 (6.5)	<0.001
Asthma	314 (3.1)	220 (2.2)	<0.001
Atrial fibrillation	1,531 (15.3)	654 (6.5)	<0.001
Cancer, lymphoma	26 (0.3)	195 (1.9)	<0.001
Cancer, metastatic	200 (2.0)	768 (7.7)	<0.001
Cancer, non-metastatic	285 (2.8)	481 (4.8)	<0.001
Chronic kidney disease	1,499 (15.0)	1,721 (17.2)	<0.001
Chronic pain	1,614 (16.1)	2,764 (27.6)	<0.001
Chronic pulmonary disease	752 (7.5)	827 (8.3)	0.052
Cirrhosis	932 (9.3)	1,019 (10.2)	0.04
Dementia	237 (2.4)	44 (0.4)	<0.001
Depression	211 (2.1)	172 (1.7)	0.05
Diabetes	354 (3.5)	409 (4.1)	0.046
Epilepsy	307 (3.1)	943 (9.4)	<0.001
Heart failure	4,945 (49.5)	2,925 (29.2)	<0.001
Hepatitis B	166 (1.7)	496 (5.0)	<0.001
Hypertension	7,132 (71.3)	7,438 (74.4)	<0.001
Hypothyroidism	235 (2.4)	916 (9.2)	<0.001
Inflammatory bowel disease	8 (0.1)	72 (0.7)	<0.001
Irritable bowel syndrome	11 (0.1)	15 (0.1)	0.556
Multiple sclerosis	14 (0.1)	49 (0.5)	<0.001
Myocardial infarction	407 (4.1)	894 (8.9)	<0.001
Parkinson's disease	243 (2.4)	164 (1.6)	<0.001
Peptic Ulcer Disease	195 (1.9)	57 (0.6)	<0.001
Peripheral Vascular Disease	3 (0.0)	1,044 (10.4)	<0.001
Psoriasis	26 (0.3)	65 (0.6)	<0.001
Rheumatoid arthritis	177 (1.8)	346 (3.5)	<0.001
Schizophrenia	171 (1.7)	93 (0.9)	<0.001
Severe constipation	447 (4.5)	182 (1.8)	<0.001
Stroke	1,577 (15.8)	1,265 (12.6)	<0.001

a *P-value indicates the statistical significance of chi-square test (or Fisher's exact test for frequencies smaller than five) comparing socio-demographics and disease prevalence between sites*.

### Comparison of Disease Dyads

**Figure 2:**
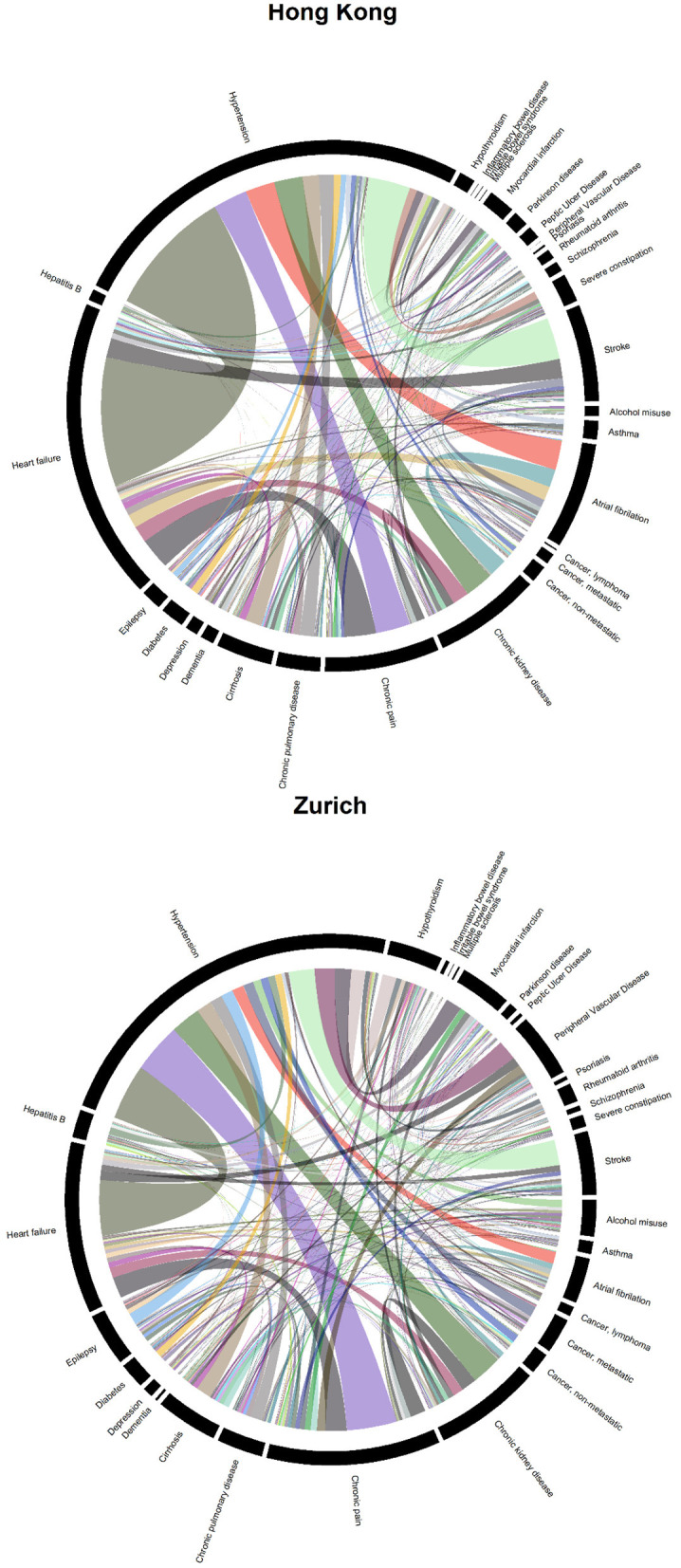
Chord diagrams showing the frequencies of disease dyads among inpatients from Hong Kong (38,964 disease dyads) and Zurich (54,052 disease dyads).

Figure 2 shows two chord diagrams which represent the frequencies of each disease dyad in both sites (represented by ribbon width). There were 54,052 co-occurring pairs of diseases in the Zurich sample but only 38,964 in the Hong Kong sample, plausibly due to the fact that patients in Zurich had more diagnoses (see Table 1). In general, the patterns of disease dyads were fairly similar. Among all dyads in Hong Kong, 10,701 were related to hypertension, 7,815 to heart failure, and 2,967 to chronic pain. In Zurich, 13,294 were related to hypertension, 6,252 to chronic pain, and 6,166 to heart failure. Patients in Zurich had more different multimorbidity patterns (more narrow ribbons): based on the presence and absence of the 30 listed diseases, there were 1,799 configurations among patients in Zurich but only 982 in Hong Kong.

### Comparison of Clusters Between Sites

**Figure 3:**
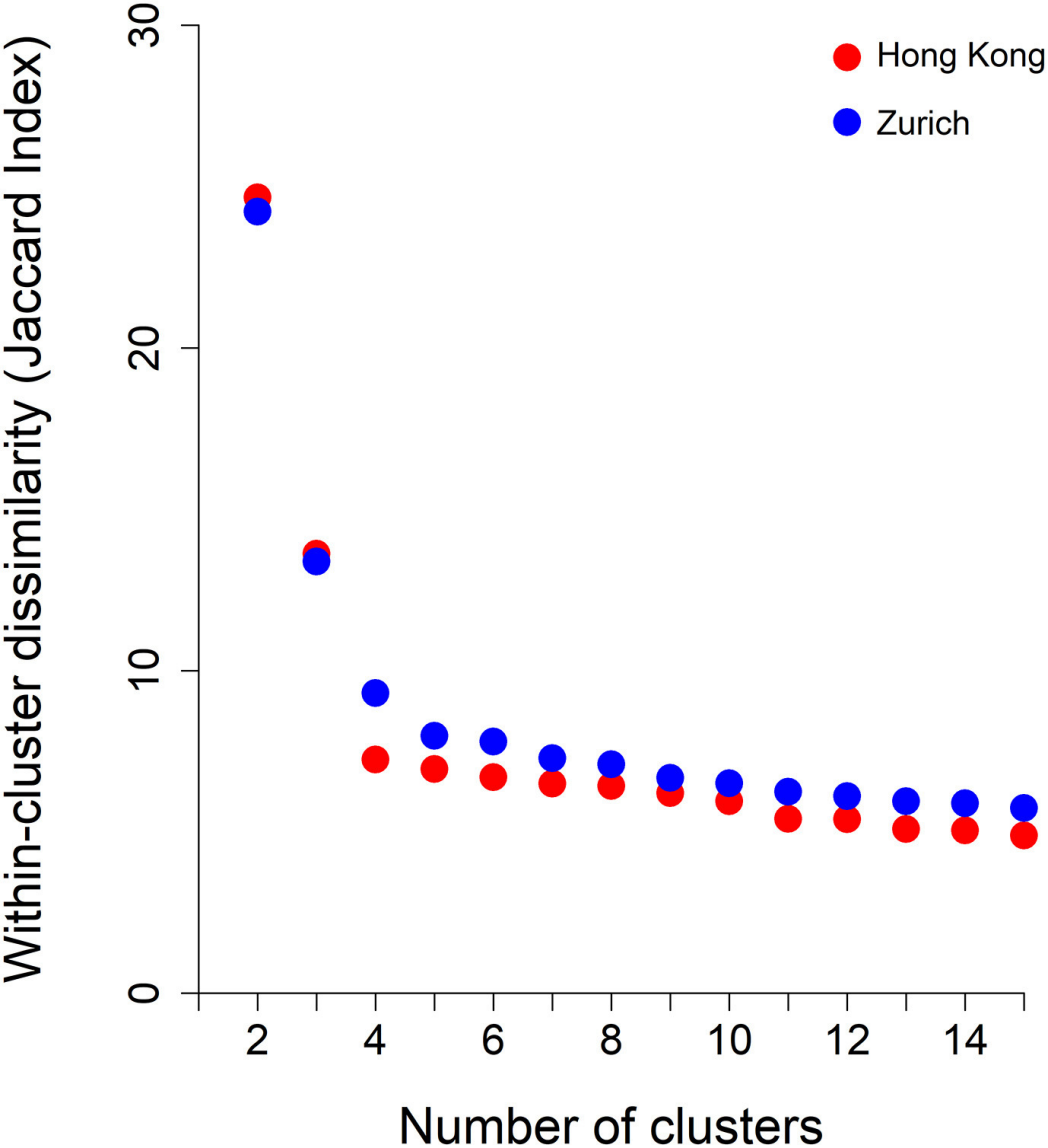
Within-cluster dissimilarity index (measured by Jaccard Index) by number of clusters derived from hierarchical agglomerative clustering and by jurisdictions, i.e., Hong Kong and Zurich.

Figure 3 shows the average within-cluster dissimilarity by number of specified clusters. Based on the elbow point method, at five clusters, within-cluster dissimilarity ceased to decline further significantly. Hence, we examined clustering schemes from 5 to 10 specified clusters in each site. This clinical review of clustering schemes indicated that at nine clusters, a balance between interpretability and meaningful categorization of patients was achieved. Thus, clustering patterns with nine specified clusters are presented. The order of clusters was in accordance with the size of the clusters (#1 being largest and #9 smallest).

**Figure 4:**
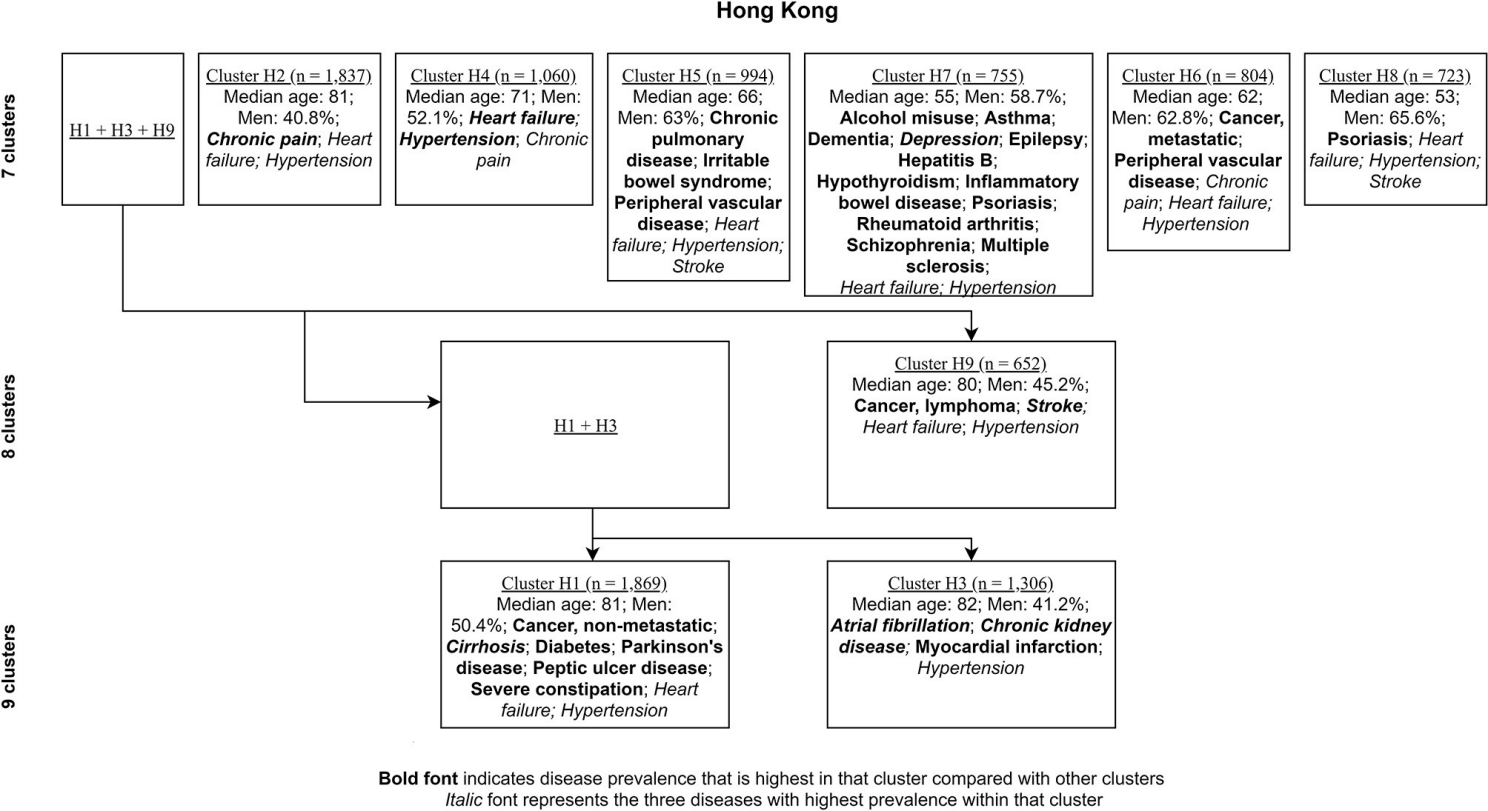
Clustering process (from seven to nine clusters) of Hong Kong inpatients labeled with characterizing diseases.

**Figure 5:**
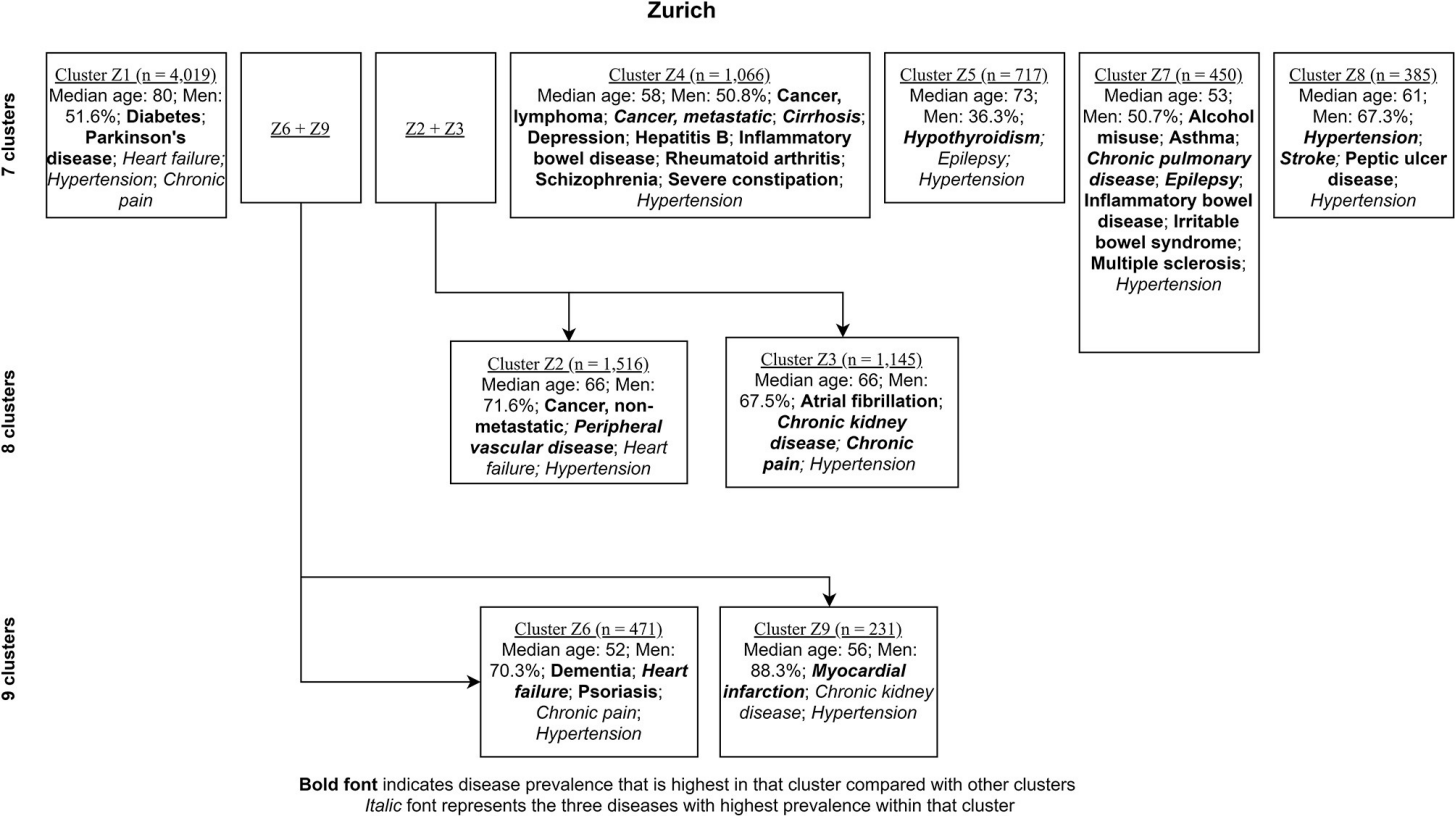
Clustering process (from seven to nine clusters) of Zurich inpatients labeled with characterizing diseases.

Figures 4, 5 show the characterizing diseases by clusters (most prevalent diseases within clusters and diseases that were most prevalent compared with other clusters) in each site respectively, while Tables 2, 3 show exact disease prevalence, median age, median length of stay, and 30-day readmission rates of clusters.

**Table 2:** Prevalence of chronic conditions by cluster (Hong Kong)^*^.

**Cluster**	**H1**	**H2**	**H3**	**H4**	**H5**	**H6**	**H7**	**H8**	**H9**
*N*	1,869	1,837	1,306	1,060	994	804	755	723	652
Age (median [IQR])	81 [76, 86]	81 [78, 86]	82 [78, 87]	71 [68, 73]	66 [63, 69]	62 [59, 64]	55 [52, 59]	53 [50, 56]	80 [76, 84]
Length of stay (median [IQR])	5 [2, 11]	4 [2, 9]	5 [2, 9]	3 [1, 6]	4 [2, 10]	3 [2, 7]	3 [1, 7]	3 [2, 7]	11 [4, 28]
30-day readmission (%)	12.3	12.1	14.7	10.3	6.6	9.6	6.6	4.7	8.3
Male (%)	50.4	40.8	41.2	52.1	63	62.8	58.7	65.6	45.2
**Chronic conditions (%)**									
Alcohol misuse	2.3	0.2	0.2	0.3	1.7	0.6	**12.5**	0.6	0.8
Asthma	9.8	0.4	0.7	1.3	0.8	0.2	**10.7**	1	0.3
Atrial fibrillation	11.2	3.2	**55.8**	1.5	30.8	2.7	7.7	1.8	18.4
Cancer, lymphoma	0.1	0.3	0.1	0.1	0.2	0.1	0.7	0.4	**0.8**
Cancer, metastatic	4.8	0.4	0.5	0.4	1.9	**5.7**	3	0.1	0.5
Cancer, non-metastatic	**9.3**	1	1.1	1.2	1.2	1.2	4.8	0.4	0.6
Chronic kidney disease	12.3	6	**61.2**	6.9	9.2	20	4.2	0.3	0.2
Chronic pain	6.4	**33.8**	8.1	27.2	3.2	28.9	5	23.1	1.8
Chronic pulmonary disease	15.6	5.7	0.7	0.4	**20.7**	3.4	12.7	0.4	1.7
Cirrhosis	**34.7**	3.8	4	1	5.9	1.1	9.3	0.8	1.2
Dementia	1.1	0.7	0.8	4.3	1.7	1.6	**12.8**	2.1	0.8
Depression	2.8	0.5	0.8	2	0.2	0.6	**14.8**	0	0
Diabetes	**13**	3.6	1.1	0.5	1	0.6	0.4	0	0.9
Epilepsy	2.4	4.2	0.6	0.8	3.4	1	**13**	2.2	1.8
Heart failure	18.4	78.4	31.2	**87.4**	35.9	76.5	22.9	67.8	29.6
Hepatitis B	1	1.1	3.6	0.7	1	1.1	**4.9**	0.8	1.7
Hypertension	59.6	82.5	72.7	**84.2**	65.9	70.5	39.3	83.3	83
Hypothyroidism	5.9	0.3	1.1	0.7	1.4	0.2	**8.6**	0.7	1.7
Inflammatory bowel disease	0	0	0	0	0.4	0	**0.5**	0	0
Irritable bowel syndrome	0.2	0	0	0.1	**0.4**	0	0	0.1	0.2
Multiple sclerosis	0.1	0	0	0.1	0.1	0.4	**0.9**	0.1	0
Myocardial infarction	0.9	0.4	**14.5**	0.8	1.9	14.1	4.4	2.2	0.5
Parkinson's disease	**8.6**	0.8	1.1	0.3	4.3	0.4	0.4	0	0.2
Peptic ulcer disease	**4.9**	0.4	1.1	2.6	1.3	1.7	2.5	0.3	0.6
Peripheral vascular disease	0	0	0	**0.1**	**0.1**	**0.1**	0	0	0
Psoriasis	0.1	0.1	0	0.5	0.3	0.4	**0.7**	**0.7**	0.3
Rheumatoid arthritis	1	3.1	0.6	0.1	1.5	0.1	**9.8**	0.1	0.2
Schizophrenia	0.4	1.9	0.3	4.9	0.8	0.2	**7.3**	0.8	0.3
Severe constipation	**13.1**	1	1.5	0.8	6.7	2.5	7.3	0	2.1
Stroke	4.6	2	4.4	7.5	39.3	1.4	3.8	35.4	**96.8**

* *Bold font denotes the cluster in which the corresponding disease is most prevalent (compared with all other clusters). Underscore denotes the most prevalent three diseases within each cluster*.

**Table 3:** Prevalence of chronic conditions by cluster (Zurich)^*^.

**Cluster**	**Z1**	**Z2**	**Z3**	**Z4**	**Z5**	**Z6**	**Z7**	**Z8**	**Z9**
*N*	4,019	1,516	1,145	1,066	717	471	450	385	231
Age (median [IQR])	80 [75, 84]	66 [61, 69]	66 [62, 70]	58 [53, 63]	73 [68, 80]	52 [49, 54]	53 [49, 57]	61 [54, 66]	56 [52, 61]
Length of stay (median [IQR])	8 [3, 14]	5 [2, 10]	7 [3, 13]	9 [4, 15]	8 [4, 14]	7 [3, 13]	8 [4, 16]	11 [7, 17]	5 [2, 9]
30-day readmission (%)	11	11.6	15.5	16	10.6	11	9.1	7	9.5
Male (%)	51.6	71.6	67.5	50.8	36.3	70.3	50.7	67.3	88.3
**Chronic conditions (%)**									
Alcohol misuse	2.3	14.6	2.5	12.2	2.5	11	**16.4**	7	3
Asthma	1.8	3.3	1.4	2.1	1.5	2.8	**6.9**	0.3	0.9
Atrial fibrillation	7.6	2.1	**20.2**	1.5	6	1.9	1.1	1.6	3
Cancer, lymphoma	1.7	0.6	1.3	**7.1**	1.5	1.5	2.2	0	0
Cancer, metastatic	7.7	1.6	1.6	**35.6**	2.8	0.6	2.2	1	0
Cancer, non-metastatic	3.6	**15.8**	1.7	3.5	4.9	0.4	0.4	0.3	0
Chronic kidney disease	19.1	3.4	**53**	2.7	10.3	25.3	3.6	4.2	18.6
Chronic pain	35.2	9.4	**50.2**	16.8	24.4	49.7	7.1	1.6	2.6
Chronic pulmonary disease	10.1	2.9	3.6	6.4	6.7	1.9	**46.9**	0.3	0.4
Cirrhosis	11.3	10.1	11.7	**19.8**	3.5	3	3.3	2.1	1.7
Dementia	0.1	0.8	0.3	0.8	0	**1.9**	1.1	0.3	0.9
Depression	0.6	3.8	0.2	**6.6**	0.4	2.1	1.1	0.3	0
Diabetes	**8.6**	0.5	1.4	0.8	3.6	0.6	0.2	0.5	0
Epilepsy	2.6	1.8	3.1	9.1	45.6	5.3	**68.7**	2.9	2.6
Heart failure	29.2	51.1	26.7	7.4	22.9	**54.8**	14.7	19	14.3
Hepatitis B	5.4	1.1	1.1	**18.3**	2	1.7	4	2.3	2.6
Hypertension	81.1	81.3	73.7	46.4	71.7	77.9	48.9	**86.2**	74.9
Hypothyroidism	4.2	1.4	3.3	17.7	**59.1**	2.5	8.7	5.2	1.7
Inflammatory bowel disease	0.5	0.6	0.9	**1.3**	0.8	0.4	**1.3**	1	0
Irritable bowel syndrome	0.1	0.1	0	0.3	0.3	0	**1.1**	0	0
Multiple sclerosis	0.2	0.4	1	0.8	0	0.2	**3.1**	0.3	0
Myocardial infarction	8.7	11.9	6.2	1.3	1.5	5.7	1.1	1.6	**98.7**
Parkinson's disease	**3.3**	0.1	0.6	0.5	2	0.4	0.4	0	0.4
Peptic ulcer disease	0.4	0.8	0.4	0.8	0.8	0.2	0.2	**1.6**	0
Peripheral vascular disease	12.8	**26.2**	5	1.9	4.3	3.6	0.4	1	0.9
Psoriasis	0.4	1.4	0.5	0.5	0.7	**1.5**	1.1	0	0.4
Rheumatoid arthritis	4.1	2.2	1.6	**8**	1.7	1.5	3.8	2.6	0
Schizophrenia	0.3	0.7	0.6	**4.1**	1.3	1.1	0.7	0.8	0
Severe constipation	1.5	0.7	0.9	**6.6**	2.2	1.1	0.9	1.3	0.4
Stroke	17.1	1.8	4.3	7.6	3.6	0.6	2.2	**98.7**	0.4

* *Bold font denotes the cluster in which the corresponding disease is most prevalent (compared with all other clusters). Underscore denotes the most prevalent three diseases within each cluster*.

**Figure 6:**
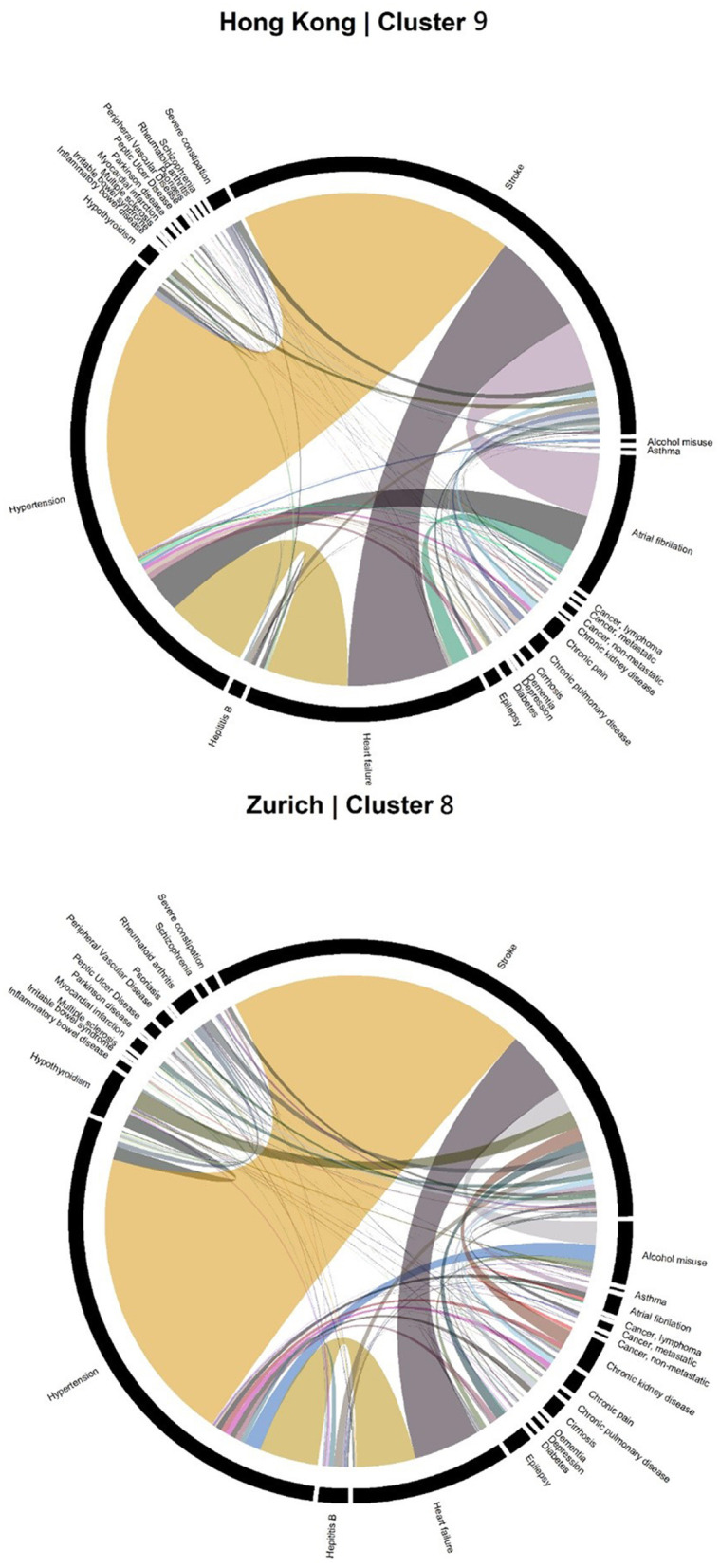
Chord diagrams showing the frequencies of disease dyads among inpatients from Cluster H9 of Hong Kong and Cluster Z8 of Zurich (stroke clusters).

**Figure 7:**
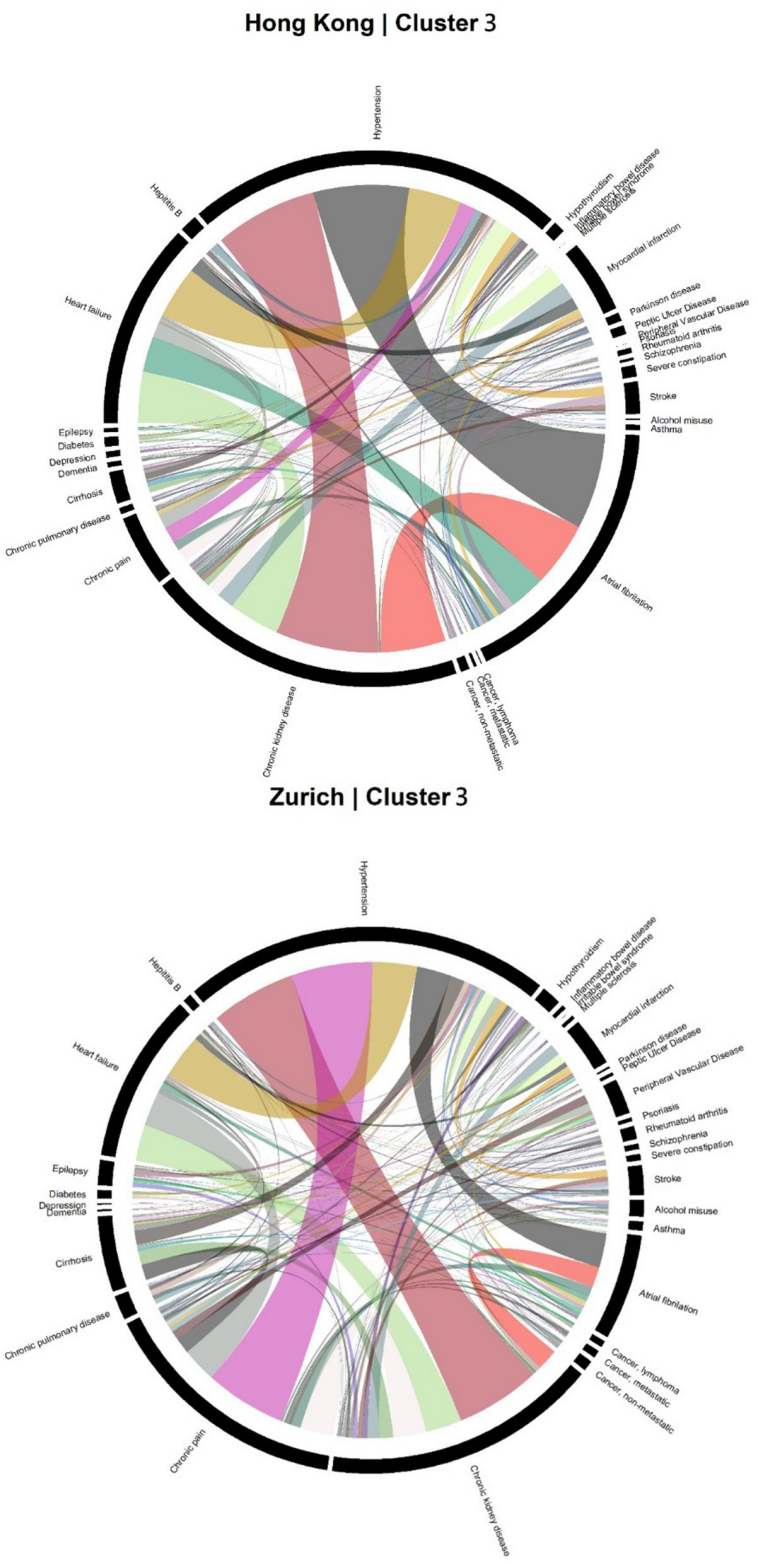
Chord diagrams showing the frequencies of disease dyads among inpatients from Cluster H3 of Hong Kong and Cluster Z3 of Zurich (chronic kidney disease clusters).

Accordingly, each site had one stroke-oriented cluster (>90% prevalence), one among older adults in Hong Kong (Hong Kong: H9, see Table 2) and another among males in Zurich (Zurich: cluster Z8, see Table 3). In both clusters, hypertension and heart failure were relatively prevalent. Figure 6 shows a comparison of the chord diagrams of these two clusters (H9 and Z8) representing all disease dyads in each site. Further, there was one chronic kidney disease-oriented cluster (>50% prevalence) in each site (H3 and Z3) and both clusters also featured atrial fibrillation (see Figure 7 for chord diagram comparison). While 53% of patients in Z3 suffered from chronic pain, H3 showed the highest prevalence of myocardial infarction across clusters in Hong Kong. The only myocardial infarction-oriented cluster in the study was generated for Zurich among relatively young males (Z9). Zurich featured two additional unique clusters, i.e., epilepsy sometimes combined with chronic pulmonary disease in young patients (Z7), and hypothyroidism in older females (Z5). Several clusters in Hong Kong featured high heart failure prevalence (H2, H6, H8, H4), and chronic pain was also relatively prevalent in those clusters. Two clusters in Zurich (Z6, Z2) showed heart failure prevalence over 50%, but only Z6 had a high proportion of chronic pain. Z2 on the other hand featured the highest prevalence of peripheral vascular disease across all clusters, whereas that diagnosis was rare in Hong Kong. Some clusters in Hong Kong and Zurich had mixed features and were thus less clear, including chronic pulmonary disease with stroke and heart failure (H5), cirrhosis with severe constipation (H1), depression, dementia, and alcohol misuse (H7), chronic pain and diabetes (Z1), as well as metastatic cancer, cirrhosis, and hepatitis B (Z4).

## Discussion

Findings of this study provide an overview of the clustering patterns of multimorbidity based on which further investigations could be conducted to inform potential integration of services and collaboration between medical specialties. In addition, the comparison between patient records from Hong Kong and Zurich provide preliminary results on the degree of universality of multimorbidity patterns across world populations. Overall speaking, there were no striking similarities or dissimilarities of the identified clustering patterns beyond established disease relationships between the two sites, with the co-occurrence of known comorbidities being most frequently observed. Specifically, only two out of nine clusters were found to be common clusters across the two sites. In both sites, especially in Hong Kong where fewer diagnoses were recorded, disease dyads mostly fell within the same medical specialties, which may suggest the importance of integrated practices within the specialty relative to cross-specialty collaboration.

### Interpretation and Implications

While a focused examination of disease prevalence within each cluster reflect only the previously identified disease relationships, the overall findings jointly represent the distribution of morbidity burden within and across clusters among multimorbid patients in a realistic healthcare setting. In other words, an overall clinical profile of multimorbid inpatients in terms of a variety of chronic conditions is presented. While the differences between the sites may be attributed to the different healthcare delivery and financing mechanisms as well as cultural and demographic factors, the identified similarities of multimorbidity patterns may suggest common specific segments of patients requiring further attention across populations, and the results convey important information on the management level for the planning and coordination of services for multimorbid patients in hospitals and other healthcare facilities. For instance, although it is commonly known that chronic kidney disease and atrial fibrillation are closely related diseases (24), our analysis further showed that patients having these two conditions constituted a significant proportion of multimorbid patients in both sites. Hence, the successful implementation of integrated care for these patients may alleviate the healthcare burden of multimorbidity significantly.

Likewise, the presence of stroke-oriented clusters which have been observed in both sites, despite having different demographics, suggest that stroke and associated morbidities (25, 26) represent a sizeable proportion of multimorbid patients in a hospital setting. While there are existing integrated services for stroke patients in typical healthcare systems of developed societies, it is also important to assess the degree to which these patients contribute to the total burden of multimorbidity.

There are also unique clusters in each site which may be of clinical importance. Specifically, only in Zurich did we observe a myocardial infarction-oriented cluster (99% prevalence). Also, the prevalence of peripheral vascular disease is drastically higher in the sample of Zurich than that of Hong Kong. These results may suggest potentially different etiologies of cardiovascular diseases between Hong Kong and Zurich due to different lifestyles, living environments, and economic structures. Nevertheless, such difference may also be attributed to different specialization foci of the hospitals, different referral patterns, and other practices that may differ between sites. More observations are needed to investigate the underlying reasons.

In Hong Kong, the clusters featuring high prevalence (>65%) of heart failure also had relatively high prevalence of chronic pain, which was partially the case in Zurich. This may relate to the regular practice of hospital clinicians in the assessment of heart failure which include the report of pain (27, 28). If confirmed by further research, this begs the question whether chronic pain prevalence is currently being underestimated among patients with other diseases (without assessment of pain) and, hence, whether it is necessary to include the assessment of pain for them. In fact, the observed prevalence of heart failure is apparently higher compared with previous inpatient research in other populations, such as in Canada (29). Further research is recommended to examine the potentially underlying reasons for this difference.

In each of the two sites, there existed highly complex clusters (H7 and Z4) in which a wide variety of diseases was featured. However, as these clusters did not constitute a markedly large proportion of multimorbid patients, integrated or collaborative care for the rest of the clusters with obvious characterizing diseases should be the priority for alleviating the healthcare burden of multimorbidity.

### Relationship With the Literature

While the results of this study are context specific, it provides preliminary information on the replicability of multimorbidity profiling between populations. In the literature, there are at least two recent important systematic reviews on the results of multimorbidity profiling from previous research with a huge variety of statistical methods (8, 30). It has been suggested that with the exceptions of mental and cardiovascular patterns, other patterns did not seem to show good evidence of universality across populations even when stratified by statistical methods of multimorbidity profiling (30). However, even with highly sophisticated meta-analytic approaches, it should be noted that across studies, different ranges of chronic conditions and specifications of statistical analyses were adopted. Therefore, evidence from analyses with the same methods on different populations is important to further our understanding. This study is one of the first attempts to narrow such a gap and as far as we are aware, this is the first study to adopt hierarchical agglomerative clustering analysis to compare multimorbid patient clustering patterns.

### Strengths and Limitations

Despite this novelty and the methodological strength of the well-validated algorithms to define chronic conditions using ICD-9 and ICD-10, this study has several limitations that require caution in interpreting the results. First, while the Hong Kong data are highly representative of the inpatients of the public sector, we do not have access to patient records in the private sector which may specialize in certain different conditions from those found prevalent in our sample. Also, University Hospital Zurich was our only source of Zurich data; nevertheless, it is a well-established general acute hospital representing diverse patient intake and standard practices. Second, the validity and comprehensiveness of diagnostic codes may differ between sites and may not be sufficiently reflective of the comparison of the true prevalence, especially considering the different healthcare financing mechanisms (social insurance in Zurich and taxation in Hong Kong), infrastructures, medical training, and practice culture, which might have considerable influence on disease coding practices (31). That might also have contributed to the rare coding of peripheral vascular disease in Hong Kong. Also, the codes were based on only one admission instead of a longitudinal assessment. Nevertheless, disease diagnoses were made by registered clinicians in each of the sites, then complemented by ICD codes given by professional teams and can therefore be reasonably considered a reliable source of disease codes in the given settings of this study. Third, inpatient data are limited to those who have already been admitted due to acute symptoms, and therefore do not represent community prevalence. For example, despite higher screening prevalence of hepatitis B in Hong Kong than Zurich (32), the observed prevalence in our Hong Kong sample is lower than that in our Zurich sample. Therefore, the findings are not readily generalizable to the outpatient populations in which multimorbid patients requiring chronic care and medications are typically better represented. Future studies are recommended to include outpatient samples with well-maintained data coding consistency and quality. Fourth, there may be under-coding of diagnoses for which no specific screening is in place upon hospital admission in general, such as mental disorders. Fifth, we only adopted a limited predetermined list of 30 diseases for the clustering analysis. Diseases not included in the list were not used for multimorbidity profiling. However, this approach allowed us to use well-validated algorithms (20) that enable first insight into the question of universality without having to validate these algorithms for the specific settings. Importantly, the present study provides preliminary evidence of some degree of universality across populations. Sixth, the comparison between the sites were mainly qualitative and no statistical tests were applied to quantitatively summarize the differences of the clustering patterns. However, our approach was a combination of clinical reasoning and exploratory machine learning methods in addressing the problem, which may facilitate future research with similar purposes. Last but not least, as the sample were randomly drawn from the Hong Kong and Zurich populations only, external generalizability is limited and the analysis should be replicated in other populations to verify the results.

To conclude, we conducted a hierarchical agglomerative clustering analysis on discharged inpatients from Hong Kong and Zurich based on a list of 30 diseases and provided findings on the universality of multimorbidity patterns across inpatient populations from the two places. Results should be facilitative of the experimentation and development of integrated or collaborative care for multimorbid patients in the healthcare systems of both populations. Future research should adopt more representative samples, longitudinal disease coding, and comprehensive lists of chronic conditions to verify results of this study and make recommendations on the care planning and coordination of services for multimorbid inpatients.

## Data Availability Statement

The data analyzed in this study is subject to the following licenses/restrictions: Authorization to access the data may be considered by the Hospital Authority of Hong Kong upon reasonable requests. Requests to access these datasets should be directed to Hospital Authority of Hong Kong, hacpaaedr@ha.org.hk.

## Ethics Statement

An ethics waiver has been granted by Cantonal Ethics Committee of Zurich for the analysis of Zurich inpatient data (Ref: NZ-B-Nr.2017-00882) while the analysis of Hong Kong inpatient data was approved by the Survey and Behavioral Ethics Committee of the Chinese University of Hong Kong (Project Code: Elderly Care – CUHK). As only secondary analysis of anonymized inpatient data was performed, no informed consent was required.

## Author Contributions

FL and PB conceptualized the study design, conducted the analysis, and drafted the manuscript. EB and SW supervised the analysis and result interpretation and critically commented on the manuscript drafts. All authors contributed to the interpretation of results and helped revise the manuscript.

## Conflict of Interest

The authors declare that the research was conducted in the absence of any commercial or financial relationships that could be construed as a potential conflict of interest.
